# Intraoperative Hemodynamic Collapse During Patent Ductus Arteriosus Ligation in an Extremely Low-Birth-Weight Infant: A Case Report

**DOI:** 10.3390/children13040518

**Published:** 2026-04-08

**Authors:** Jeongsoo Choi, Ho Soon Jung, Da Hyung Kim, Yong Han Seo, Hea Rim Chun, Hyung Yoon Gong, Jae Young Ji, Jin Soo Park, Sangwoo Im

**Affiliations:** Department of Anesthesiology and Pain Medicine, Cheonan Hospital, College of Medicine, Soonchunhyang University, Cheonan 31151, Republic of Korea; 124393@schmc.ac.kr (J.C.); dyflam@schmc.ac.kr (H.S.J.); 121826@schmc.ac.kr (D.H.K.); c75501@schmc.ac.kr (Y.H.S.); 98303@schmc.ac.kr (J.Y.J.); 118541@schmc.ac.kr (J.S.P.); 141990@schmc.ac.kr (S.I.)

**Keywords:** patent ductus arteriosus, extremely low birth weight infant, post-ligation cardiac syndrome

## Abstract

**Highlights:**

**What are the main findings?**

- In an extremely low birth weight infant, abrupt intraoperative circulatory collapse during PDA ligation showed features resembling the pathophysiologic mechanism of post-ligation cardiac syndrome.
- A concomitant left-sided pneumothorax, identified postoperatively, likely aggravated the intraoperative hemodynamic instability by worsening oxygenation and reducing venous return.

**What are the implications of the main findings?**

- PLCS-like afterload mismatch may manifest as intraoperative hemodynamic collapse, not only as a delayed postoperative complication, in extremely low birth weight infants undergoing PDA ligation.
- Recognizing this possibility and carefully monitoring for pneumothorax and ventilation-related factors may be important for anticipatory hemodynamic management in extremely low birth weight infants undergoing PDA ligation.

**Abstract:**

**Background and Clinical Significant:** Patent ductus arteriosus (PDA) is a common cardiovascular disorder in extremely low-birth-weight (ELBW) infants, for which surgical ligation is indicated when pharmacologic closure fails. Sudden increases in afterload combined with immature myocardial contractility can lead to post-ligation cardiac syndrome (PLCS), which usually occurs within hours after surgery. However, acute intraoperative hemodynamic collapse during PDA ligation has rarely been described. **Case Presentation:** A preterm infant born at 24 weeks and 3 days of gestation with a birth weight of 890 g underwent emergency PDA ligation for a hemodynamically significant PDA (hs-PDA) refractory to pharmacological treatment. Fifteen minutes after skin incision, the infant developed desaturation, bradycardia, and non-measurable noninvasive blood pressure, which required immediate hemodynamic resuscitation with manual ventilation, fluid administration, and dopamine and dobutamine infusions. Hemodynamics gradually recovered after completion of ductal ligation, whereas oxygen saturation did not fully recover. Postoperative chest radiography revealed a left-sided pneumothorax, and oxygen saturation stabilized after pleural air aspiration. The subsequent clinical course was uneventful, and typical PLCS did not develop. **Conclusions:** This case suggests that intraoperative hemodynamic collapse during PDA ligation may share pathophysiologic features with PLCS, and that concomitant pneumothorax can further aggravate hemodynamic instability by worsening hypoxemia and reducing venous return.

## 1. Introduction

Patent ductus arteriosus (PDA) is a common cardiovascular complication in extremely low-birth-weight (ELBW) infants. Wani et al. reported that the incidence of PDA was 56.2% in preterm infants with birthweights below 1000 g and 50% in those with gestational ages less than 30 weeks [1,2]. However, not all PDAs are clinically significant, and determining the hemodynamic relevance of the ductus remains a key challenge in neonatal care. Beyond simple ductal diameter, contemporary neonatal functional echocardiography frameworks define a hs-PDA using a multiparametric approach that integrates ductal characteristics, pulmonary overcirculation, and systemic hypoperfusion. In practice, echocardiographic assessment integrates ductal size and shunt pattern with indices of left-sided volume loading, such as an increased left atrial-to-aortic root ratio and left ventricular dimensions. These findings are interpreted alongside clinical signs of end organ hypoperfusion, including oliguria, metabolic acidosis, and escalating respiratory support [3,4]. Pharmacological management with indomethacin or ibuprofen is typically used as first-line therapy [5]. In addition, recent evidence suggests that, in ELBW infants with hs-PDA, a second course of medical therapy using ibuprofen or acetaminophen after failure of the first course can achieve ductal closure in a substantial proportion of cases, which may reduce the need for surgical intervention [6]. However, surgical ligation is required in patients who fail medical treatment or present with contraindications such as bleeding diathesis or necrotizing enterocolitis [7,8]. The optimal timing of surgical ligation remains controversial in preterm and ELBW infants. A recent systemic review suggests that earlier ligation may provide short-term respiratory and nutritional benefits; however, its effects on mortality and major morbidities remains inconclusive [9]. Although surgical ductal ligation is an effective and commonly used method for PDA closure, it may be associated with various hemodynamic complications [5,10]. Among these, post-ligation cardiac syndrome (PLCS) is the most prominent [2]. PLCS typically occurs within hours after surgery and is characterized by hypotension and impaired cardiac function resulting from an abrupt increase in systemic vascular resistance combined with immature myocardial contractility [2]. While postoperative complications after PDA ligation are relatively well documented, intraoperative hemodynamic instability has been rarely reported. In ELBW infants, early recognition and mechanistic interpretation of these events are particularly challenging due to limited hemodynamic monitoring and immature cardiovascular regulatory mechanisms. Therefore, understanding the clinical significance and pathophysiology of acute circulatory collapse during PDA ligation is important. This case report describes an episode of sudden hemodynamic collapse during PDA ligation in an 890 g preterm infant. Although typical PLCS did not develop postoperatively, the clinical features observed intraoperatively were consistent with the pathophysiology of PLCS, namely acute increases in afterload in the setting of limited myocardial reserve. This case highlights the importance of recognizing anesthetic considerations and clinical implications of intraoperative hemodynamic instability in ELBW infants undergoing PDA ligation.

## 2. Case Presentation

**Figure 1:**
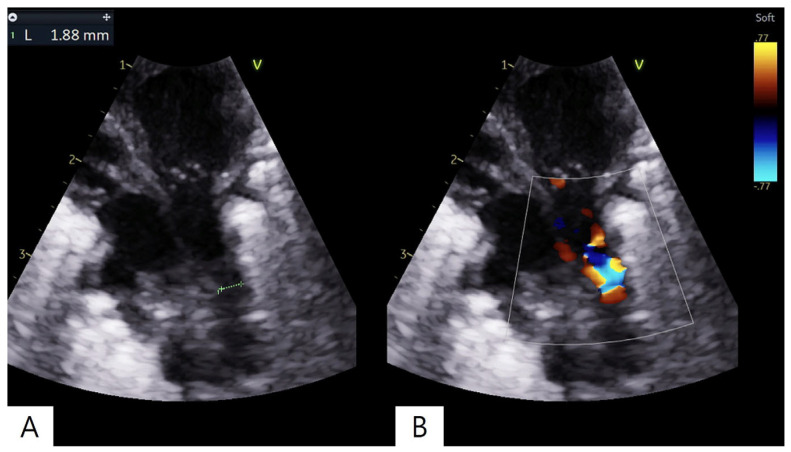
Transthoracic echocardiographic image obtained on postnatal day 8 demonstrating a hemodynamically significant patent ductus arteriosus. (**A**) Two-dimensional echocardiography shows a ductal diameter of approximately 1.8 mm. (**B**) Color Doppler imaging demonstrates bidirectional shunt flow across the ductus arteriosus. Numbers 1–3 on the left margin indicate depth markers of the ultrasound image, and the dotted green line represents the measured ductal diameter.

**Figure 2:**
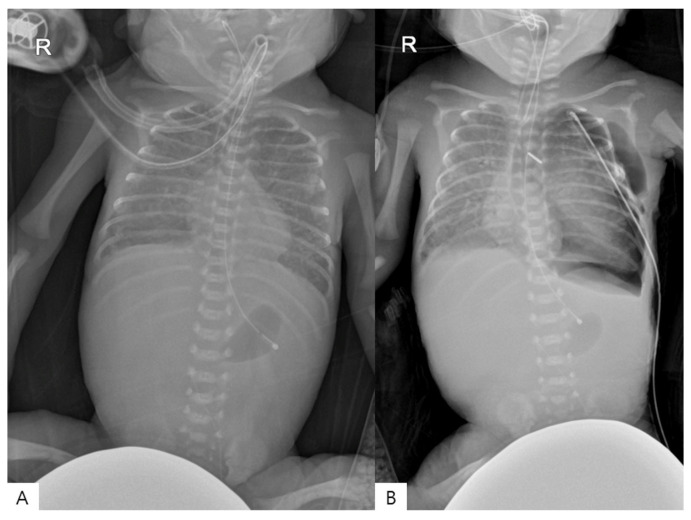
Preoperative and postoperative anteroposterior chest and abdominal radiograph. (**A**) Preoperative image shows diffuse bilateral lung haziness without pneumothorax. (**B**) Postoperative image reveals a left-side pneumothorax. R indicates the right side.

A male ELBW infant was born at a gestational age of 24 weeks and 3 days with a birth weight of 890 g. Immediately after birth, he was admitted to the neonatal intensive care unit (NICU) due to severe perinatal asphyxia, presenting with a heart rate 50 bpm and an oxygen saturation of 20%. The infant was intubated and received prophylactic surfactant therapy with poractant alfa (Curosurf^®^, Chiesi Farmaceutici, Parma, Italy), administered at a dose of 200 mg/kg via the endotracheal tube within 10 min of birth. Mechanical ventilation was subsequently initiated in assist-control mode with the following settings: fraction of inspired oxygen(FiO_2_) 0.8, respiratory rate 50 breaths/min, peak inspiratory pressure 20 cmH_2_O, and positive end-expiratory pressure 6 cmH_2_O. Transthoracic echocardiography performed on postnatal day 2 revealed a PDA measuring 0.93 mm. Serial echocardiographic assessments were performed by a neonatal intensive care specialist on seven occasions, with one additional evaluation by a cardiologist. The evaluations consistently included ductal characteristics (diameter and shunt direction), ventricular function, and LA/Ao ratio, along with associated cardiac findings. The detailed clinical course during the first 7 postnatal days, including echocardiography, urine output, and serial arterial blood gas analyses, is summarized in Table 1. Arterial blood gas analyses were performed multiple times per day as clinically indicated, and representative values are presented. During this period, persistent metabolic acidosis was noted, and the PDA progressively increased in size despite medical management. Subsequently, the patient exhibited clinical deterioration, including a decrease in urine output to 1.0 cc/kg/h. Follow-up echocardiography on postnatal day 8 revealed a ductal diameter of 1.8 mm with bidirectional shunting and an aorta–left pulmonary artery pressure gradient of 15 mmHg, along with findings suggestive of left-sided volume overload (Figure 1). The patient was diagnosed with a hsPDA, and emergency PDA ligation was planned. The diagnosis of hemodynamically significant PDA was based on combined echocardiographic and clinical criteria, including progressive ductal enlargement with bidirectional shunting and evidence of systemic hypoperfusion, such as decreased urine output and persistent metabolic acidosis, despite medical treatment. Upon arrival in the operating room, the patient remained intubated and was transported with manual ventilation using a bag-valve device. Anesthesia was induced with sevoflurane 3.0% and rocuronium 0.5 mg. Considering the infant’s small size, a neonatal blood pressure cuff was placed on a finger and a pulse oximetry probe was attached to the palm for monitoring. Surgery was performed via posterolateral thoracotomy in the right lateral decubitus position. At the start of surgery, vital signs were stable, with a blood pressure of 42/18 mmHg, heart rate of 158 beats per minute, and oxygen saturation of 98%. Fifteen minutes after the start of surgery, oxygen saturation abruptly decreased to 77%. Although FiO_2_ was increased to 0.7, oxygen saturation further declined to 35%. Simultaneously, noninvasive blood pressure (NIBP) became unmeasurable, and the heart rate dropped to 45 beats per minute, indicating severe bradycardia and hypotension. FiO_2_ was increased to 1.0, and manual ventilation was resumed, resulting in improvement of oxygen saturation to 95%. However, NIBP remained unmeasurable, and bradycardia persisted for up to 12 min following the event, although electrocardiography demonstrated a normal sinus rhythm throughout. Dobutamine and dopamine (prepared as 1.47 mL diluted in 50 cc) were initiated at doses of 5 µg/kg/min each, and intravenous fluid administration (1:4 mixture of 5% dextrose and normal saline) was increased to 10 cc/h. A profound hemodynamic collapse, characterized by the inability to measure blood pressure, persisted for approximately 20 min. Immediately after completion of PDA ligation by the surgeon, blood pressure gradually recovered to 41/17 mmHg, and heart rate improved to 132 bpm. Despite hemodynamic recovery, oxygen saturation did not improve adequately with mechanical ventilation. Therefore, manual ventilation with FiO_2_ 1.0 was continued until the end of surgery, maintaining oxygen saturation at approximately 95%. Neuromuscular blockade was reversed using pyridostigmine, and spontaneous respiration returned; however, adequate oxygen saturation could not be maintained without manual ventilation. The patient was transferred to the NICU while continuing manual ventilation. Upon arrival in the NICU, high-frequency oscillatory ventilation was initiated with FiO_2_ 1.0, frequency 13 Hz, mean airway pressure 13 cmH_2_O, and amplitude 30. Vital signs at that time were as follows: blood pressure 36/11 mmHg, heart rate 159 beats per minute, respiratory rate 60 breaths per minute, oxygen saturation 90%, and body temperature 36 °C. A subsequent chest radiograph confirmed a left pneumothorax (Figure 2). After aspiration of 30 cc of air through the inserted chest tube, oxygen saturation normalized to 99%. The patient showed initial improvement, with blood pressure around 54/28 mmHg and heart rate at 155 beats per minute. However, the patient required ongoing respiratory and hemodynamic support, and gradual stabilization was achieved over time as the pneumothorax resolved. Follow-up echocardiography confirmed successful closure of the PDA. No further episodes of hemodynamic deterioration attributable to the PDA or ductal ligation were observed thereafter, and there was no clinical evidence suggestive of PLCS during the postoperative period.

## 3. Discussion

This case describes an episode of acute hemodynamic collapse occurring during PDA ligation in an ELBW infant with a birth weight of 890 g. Hemodynamic deterioration following PDA ligation is classically recognized as PLCS, which typically occurs within the first 6–24 h after surgery and is characterized by hypotension, low cardiac output, and impaired oxygenation [2]. Sehgal et al. reported hemodynamic instability, including hypotension and low cardiac output, occurring after PDA ligation [11]. Although their case demonstrated similar underlying pathophysiology, in our case the hemodynamic collapse occurred abruptly during the ligation procedure.

PLCS refers to a hemodynamic state characterized by an abrupt increase in left ventricular afterload following ductal closure, along with the loss of left-to-right shunting that previously contributed to systemic cardiac output. These changes result in a sudden rise in systemic vascular resistance and a reduction in pulmonary blood flow, leading to decreased left ventricular preload. Consequently, cardiac output may decline, predisposing affected infants to hypotension and low cardiac output syndrome [12]. Thus, PDA ligation promptly relieves pulmonary overcirculation while simultaneously imposing an abrupt hemodynamic burden on the left ventricle [13]. The most critical pathophysiological mechanism underlying this process is left ventricular afterload mismatch [14]. In the early postnatal period, when the ductus arteriosus remains patent, the left ventricle ejects a substantial portion of its output into the low-resistance pulmonary circulation and consequently adapts to a relatively low afterload environment. However, following surgical ligation, the elimination of this low-resistance shunt abruptly exposes the left ventricle to a markedly increased systemic vascular resistance [12]. The immature myocardium of preterm infants has limited capacity to compensate for acute increases in afterload, which can result in reduced cardiac output and subsequent hemodynamic instability. The present case demonstrates that hemodynamic alterations induced by PDA ligation may manifest as immediate intraoperative circulatory collapse without a delayed onset. This finding suggests that, in ELBW infants undergoing PDA ligation, hemodynamic instability is not confined to the postoperative period but may also occur during the intraoperative phase. In addition to the hemodynamic alterations described above, intraoperative vagal stimulation represents another plausible mechanism contributing to the cardiovascular instability observed in this case. Anatomically, the ductus arteriosus lies in close proximity to the vagus nerve and the left recurrent laryngeal nerve [15]. During surgical exposure and ligation of the ductus, direct traction or manipulation of these structures may provoke a pronounced parasympathetic reflex response, resulting in bradycardia [15,16]. ELBW infants are particularly vulnerable to such vagal responses due to immaturity of autonomic nervous system regulation, which may amplify the severity of vagally mediated bradycardia. In this case, the severe hypoxemia that accompanied the hemodynamic collapse was likely associated with the pneumothorax identified postoperatively. Although classical PLCS has been described as a delayed postoperative phenomenon, the underlying physiology may not necessarily require a latency of several hours. Once the ductus is effectively occluded, the left ventricle is immediately exposed to the full systemic afterload while simultaneously losing the low-resistance runoff pathway, so that a sudden decline in cardiac output may occur as soon as ductal flow is abolished in an immature myocardium with limited contractile reserve. In ELBW infants, even a brief period of afterload-preload mismatch may be sufficient to precipitate abrupt hypotension, particularly when combined with anesthetic induced vasodilation, mechanical impairment of venous return due to thoracotomy and positive pressure ventilation, and additional respiratory compromise, including pneumothorax, as observed in this case. We therefore consider that this episode may represent an intraoperative manifestation of the same afterload mismatch mechanism that underlies PLCS, rather than a deviation from the classical timing based definition of PLCS.

The exact timing of pneumothorax development could not be clearly determined. Approximately 15 min after the start of surgery, both blood pressure and oxygen saturation decreased simultaneously during surgical exposure of the ductus. This period of hemodynamic and respiratory instability persisted for approximately 20 min. Following completion of ligation, blood pressure improved, whereas oxygen saturation did not fully recover. During PDA ligation, transient lung collapse or entry of air into the pleural space may occur as part of surgical exposure, and this is typically resolved by the end of the procedure. However, in this case, the persistence of hypoxemia despite completion of the surgery suggests that a clinically significant pneumothorax may have developed rather than a transient intraoperative phenomenon. This temporal pattern suggests that the initial event was likely related to combined hemodynamic and respiratory compromise during surgical manipulation. In extremely low-birth-weight infants, lung retraction and changes in intrathoracic pressure during thoracic procedures may significantly affect both ventilation and venous return. The subsequent recovery of blood pressure after ligation indicates partial resolution of the circulatory component, while the persistent impairment in oxygenation raises the possibility of a superimposed respiratory complication. Pneumothorax occurring during PDA ligation can increase intrathoracic pressure, thereby impairing venous return and exacerbating hypotension [17]. In this patient, oxygen saturation could not be restored with mechanical ventilation but was temporarily maintained with manual ventilation, suggesting that the reduced lung compliance caused by pneumothorax was transiently overcome by the application of higher airway pressures. However, such high-pressure ventilation may have further compromised venous return, thereby reducing preload and potentially exacerbating the hemodynamic instability in the setting of left ventricular afterload mismatch. This may explain the prolonged period during which blood pressure could not be measured. In addition, forced lung expansion during manual ventilation may have contributed to the development or aggravation of a delayed pneumothorax, which could explain the persistent difficulty in maintaining adequate oxygen saturation after arrival in the NICU. The subsequent stabilization of oxygen saturation following aspiration of 30 mL of air through the chest tube suggests that the hemodynamic instability observed in this case resulted from a complex interplay between cardiac dysfunction related to left ventricular afterload mismatch and a surgical complication in the form of pneumothorax.

Several conditions may mimic PLCS, including sepsis, hypovolemia, adrenal insufficiency, pulmonary hypertensive crisis, and residual ductal patency [11,18]. In the present case, these possibilities were considered. However, there were no clinical features strongly suggestive of sepsis. Although leukocytosis was observed during the clinical course, it was not accompanied by other clinical signs consistent with infection, and serum lactate levels remained within the normal range. In addition, platelet counts remained within an acceptable range, making severe systemic infection less likely. Echocardiography did not demonstrate residual ductal patency or findings suggestive of pulmonary hypertensive crisis. Hypovolemia and adrenal insufficiency were considered less likely based on the overall perioperative course and the absence of findings strongly supporting these conditions.

Although NIBP could not be measured for approximately 20 min, this period was not interpreted as a state of complete cardiac arrest. Bradycardia was observed. However, continuous electrocardiographic monitoring demonstrated a persistent normal sinus rhythm, indicating preserved electrical cardiac activity. In addition, the possibility of transient measurement failure related to the use of a finger-mounted blood pressure cuff could not be excluded. Considering the surgeon’s intraoperative observation of ongoing cardiac activity, the event in this case was more likely attributable to a transient hemodynamic disturbance occurring during ductal ligation rather than irreversible circulatory arrest. Accordingly, dopamine and dobutamine were administered to provide immediate support for the acute hypotension and reduced cardiac output observed during the ligation process. In PLCS, milrinone, a phosphodiesterase type 3 inhibitor, is commonly used to mitigate left ventricular dysfunction and afterload mismatch. Milrinone reduces systemic afterload, enhances cardiac output, and improves myocardial relaxation, thereby facilitating hemodynamic recovery [11]. In situations such as the present case, where acute circulatory collapse occurs intraoperatively, the vasodilatory effects of milrinone must be considered because of the potential risk of exacerbating hypotension [19]. Therefore, catecholamine-based inotropic and vasopressor agents were administered as first-line therapy to achieve rapid stabilization of systemic perfusion.

This case has several limitations. First, invasive arterial blood pressure monitoring was not performed, limiting precise quantification of intraoperative hypotension. In ELBW infants, placement of an arterial line can be technically challenging and is not always routinely performed. Continuous invasive monitoring could have enabled earlier detection of rapid hemodynamic changes and provided more accurate guidance for the management of circulatory instability. Second, the absence of serial biochemical measurement during the intraoperative period precluded objective confirmation of hypoxia. Instead, oxygenation status was assessed using continuous pulse oximetry. Third, the exact timing of pneumothorax development during surgery could not be clearly determined, as it was identified postoperatively. Manual ventilation administered to manage hypoxemia secondary to pneumothorax may have increased intrathoracic pressure and reduced venous return, which might have contributed to the prolonged hypotensive state. Although the presence of pneumothorax prior to ligation cannot be completely excluded, the clinical course makes this less likely. The patient remained relatively stable during the initial phase of surgery, and both blood pressure and oxygen saturation decreased abruptly approximately 15 min after surgical exposure. In addition, the recovery of blood pressure following ligation suggests that the primary event involved a hemodynamic component. The persistence of hypoxemia despite circulatory improvement raises the possibility that pneumothorax became clinically significant during or after this period rather than being the initial cause of deterioration. Fourth, as this is a single case report, there are inherent limitations in establishing a definitive causal relationship among ductal ligation, pneumothorax, and hemodynamic collapse.

## 4. Conclusions

PLCS has traditionally been recognized as a postoperative complication occurring within hours after PDA ligation. However, this case suggests that the same underlying pathophysiology, an abrupt increase in left ventricular afterload in the setting of limited myocardial reserve, may manifest as immediate circulatory collapse during the ligation process itself. This observation highlights the need to recognize that PLCS-like hemodynamic collapse may occur not only in the postoperative period but also during intraoperative management in ELBW infants.

## Figures and Tables

**Table 1:** Serial echocardiographic and clinical findings during the first 8 postnatal days. Arterial blood gas values represents clinically relevant measurements for each day.

Postnatal Day	PDA Diameter (mm)	Shunt Direction	LA/Ao Ratio	pH	Base Excess	Urine Output (mL/kg/h)	Intervention
2	0.93	Left to Right	0.72	7.21	−7.5	4.0	−
3	1.58	Bidirectional	1.22	7.19	−9.8	3.8	−
4	1.66	Bidirectional	1.23	7.17	−8.6	3.9	−
5	1.66	Bidirectional	1.39	7.19	−8.0	4.0	Ibuprofen
6	1.11	Bidirectional	1.28	7.18	−9.3	4.0	Ibuprofen
7	1.18	Bidirectional	0.95	7.19	−7.1	3.0	Ibuprofen
8	1.80	Bidirectional	1.42	7.22	−8.9	1.0	Surgical ligation

## Data Availability

No new data were created or analyzed in this study.

## Abbreviations

PDA	Patent ductus arteriosus
ELBW	Extremely low birth weight
hs-PDA	Hemodynamic significant patent ductus arteriosus
PLCS	Post-ligation cardiac syndrome
FiO_2_	Fraction of inspired oxygen
NIBP	Noninvasive blood pressure
NICU	Neonatal intensive care unit
